# Male-Biased Genes in Catfish as Revealed by RNA-Seq Analysis of the Testis Transcriptome

**DOI:** 10.1371/journal.pone.0068452

**Published:** 2013-07-12

**Authors:** Fanyue Sun, Shikai Liu, Xiaoyu Gao, Yanliang Jiang, Dayan Perera, Xiuli Wang, Chao Li, Luyang Sun, Jiaren Zhang, Ludmilla Kaltenboeck, Rex Dunham, Zhanjiang Liu

**Affiliations:** The Fish Molecular Genetics and Biotechnology Laboratory, Department of Fisheries and Allied Aquacultures and Program of Cell and Molecular Biosciences, Aquatic Genomics Unit, Auburn University, Auburn, Alabama, United States of America; University of California, Davis, United States of America

## Abstract

**Background:**

Catfish has a male-heterogametic (XY) sex determination system, but genes involved in gonadogenesis, spermatogenesis, testicular determination, and sex determination are poorly understood. As a first step of understanding the transcriptome of the testis, here, we conducted RNA-Seq analysis using high throughput Illumina sequencing.

**Methodology/Principal Findings:**

A total of 269.6 million high quality reads were assembled into 193,462 contigs with a N50 length of 806 bp. Of these contigs, 67,923 contigs had hits to a set of 25,307 unigenes, including 167 unique genes that had not been previously identified in catfish. A meta-analysis of expressed genes in the testis and in the gynogen (double haploid female) allowed the identification of 5,450 genes that are preferentially expressed in the testis, providing a pool of putative male-biased genes. Gene ontology and annotation analysis suggested that many of these male-biased genes were involved in gonadogenesis, spermatogenesis, testicular determination, gametogenesis, gonad differentiation, and possibly sex determination.

**Conclusion/Significance:**

We provide the first transcriptome-level analysis of the catfish testis. Our analysis would lay the basis for sequential follow-up studies of genes involved in sex determination and differentiation in catfish.

## Introduction

Sex is one of the most fundamental features of life. In teleost, sex determination mechanism exhibits extraordinary plasticity and diversity during evolution. Fish represent over 50% of all vertebrates with over 32,400 species. The vast majority of fish species are gonochoristic although some fish are hermaphroditic. The latter is unique in fish among vertebrates [1], [2]. With gonochoristic fishes, the sex could be either genetic sex determination (GSD) or environmental sex determination (ESD), and sometimes a combination of both. In most cases among fishes, sex is determined by GSD, but environmental factors, especially temperature, can influence sex determination [3].

The most common sex determination system in fish is the XY system, i.e., male heterogamety. However, in many species, the sex determination system is the ZW system, i.e., female heterogamety. For instance, medaka (*Oryzias latipes*) [4] and rainbow trout (*Oncorhynchus mykiss*) [5] have XY sex determination system, while turbot (*Scophthalmus maximus*) [6] and half-smooth tongue sole (*Cynoglossus semilaevis*) [7] have ZW sex determination system. Even in closely related species, the sex determination system may vary. For example, in tilapia, it is believed that both XY and ZW systems are functional depending on the species [3]. Due to the unrivaled diversity, the study of sex determination in fish becomes a very intriguing research area. Such studies have demonstrated unprecedented diversity. However, to date, sex determination mechanisms have been elucidated only in five fish species including medaka (*O. latipes*) [4], rainbow trout [5], fugu [8], Patagonian pejerrey (*Odontesthes hatcheri*) [9] and another species of medaka (*O. luzonensis*) [10]. In medaka (*O. latipes*), DMY gene was found to be the sex determination gene, which is a duplicated gene of Dmrt1 on the Y-chromosome. In Fugu, there was no Y-specific gene that controls sex. Instead, a single nucleotide polymorphism within the anti-Mullerian hormone receptor type II (Amhr2) gene was found to control the sex [8]. In rainbow trout, SDY gene, a truncated immune related gene interferon regulatory factor 9 like gene residing on the Y-chromosome, was found to be the sex control gene. With Patagonian pejerrey, Amhy gene, which is a male-specific, functional duplicated copy of the anti-Mullerian hormone gene (*amh*) was suggested as a strong candidate of the master sex-determining gene in this species [9]. In addition to these where the sex determination gene has been identified, active research is underway with a number of aquaculture fish species because of potential applications of sex determination mechanisms for aquaculture production [7], [11]–[15].

Channel catfish (*Ictalurus punctatus*) is the most important aquaculture species in the United States, and also an excellent model organism for teleost genetics and genomics studies [16]. Sex in catfish is mainly determined by the genetic sex determination (GSD) system, with the co-existence and interaction with temperature-dependent sex determination (TSD) [17]. Genetically, catfish possess XY male heterogametic system. Although the sex differentiation was well documented for channel catfish [17], [18], little is known for its sex-determining genes.

Two major approaches have been used for studies of sex-determining genes in teleost fish (using XY system as an example): 1) Identification of Y-specific sequences; 2) Identification of male-specific transcripts. In both cases, the sex-determining gene must be validated by functional analysis of the Y-specific sequences and male specific transcripts for their necessity and sufficiency for sex determination. Apparently, when the Y-chromosome is highly divergent from the X chromosome, the first approach is quite advantageous. However, no significant difference in DNA content was detected from male and female cells in channel catfish, which is consistent with the reports in many other fish species in which the karyotype of X chromosome and Y chromosome is very similar [19]. In such cases, male and female genomes carry identical or almost identical DNA. It is possible that the subtle differences of a very small portion of genes located on sex-specific chromosomes or sex-determining region are responsible for the sex determination [20]–[22], and discovery of the Y-specific sequences may be difficult. In the extreme situation under this case like in the case of Fugu, there are no Y-specific sequences or Y-specific transcripts. Instead, only a single SNP is responsible for sex determination. Therefore, the study of sex determination gene can then be extremely difficult. Apparently, analysis of male-specific transcripts could provide some advantage than analysis for Y-specific sequences in the absence of a whole genome sequence. One possibility does exist, i.e., some genes may be expressed only in the males, but not in the females. Obviously, testis is a male organ and therefore transcriptome analysis using testis tissue could be of interest for the potential detection of male-biased transcripts, which may or may not involve sex determination gene(s).

Nevertheless, analysis of genes expressed in male-specific organs has been utilized as one of the approaches to identify male-specific transcripts. Sex-biased genes expressed predominantly or exclusively in one sex were thought to drive the phenotypic differences between males and females [20], [23]. It was reported that in adult zebrafish the difference of the key transcript abundance between males and females may cause the phenotypic sexual dimorphism mediated by sex differences in gene expression, and these differences likely play a major role in phenotypic sexual dimorphism [24]. A large fraction of the expression divergence between sexes occurs in the gonads [25], and the sex-biased genes are expected to exhibit a higher possibility to be male-specific genes involved in sex determination [26].

In catfish, we have previously made major efforts for transcriptome analysis. Early efforts used EST analysis [27]–[32], and such collective efforts allowed identification of around 500,000 ESTs in catfish. Recently, the application of the nextgen sequencing technologies allowed identification of over 25,000 genes in catfish through RNA-Seq [33], [34]. Such efforts have led to sequencing as well as assembly of over 14,000 full length cDNAs [33], [35]. However, in all such transcriptome analysis, no testis tissues were used, and therefore, genes specifically or preferentially expressed in the testis could have been missed. As a part of the catfish genome program to provide expression evidence for gene models for genome assembly and annotation, the major objective of this study was to generate additional transcriptome information from the testis tissue. In this study, we took advantage of the high throughput next generation sequencing and conducted RNA-Seq of the testis tissue with a goal of profiling the genes expressed in the testis. Because testis is a male specific organ, our additional interest is to identify male-biased transcripts, which could contribute to our ongoing efforts to elucidate sex determination mechanisms in catfish.

## Results

### Sequencing and Assembly of Short Expressed Reads from Catfish Testis

A total of 294.6 million reads of 100 bp long were generated. Ambiguous nucleotides, low-quality sequences (quality scores <20) and short reads (length <30 bp) were removed, and the remaining high-quality reads (91.5%) were carried forward for transcriptome assembly and analysis (Table 1).

**Table 1:** Summary of RNA-Seq of the testis transcriptome.

Name	Number
Number of reads from raw data	294,618,596
Average read length (bp)	100
Number of reads after trimming	269,572,695
Percentage retained	91.5%
Average read length after trimming (bp)	93.9

ABySS & Trans-ABySS was used for the assembly of the RNA-Seq short reads because they provided a superior assembly when compared with Velvet and CLC Genomics Workbench [36]. Use of Trans-ABySS to merge ABySS multi-k-assembled contigs resulted in an initial approximately 6.0 million contigs with average length of 129.5 bp and N50 size of 163 bp. A total of 340,000 contigs with lengths greater than 200 bp were carried forward for additional analysis. CAP3 was employed to remove redundancy generated during multi-k assemblies. Following CAP3, 193,462 contigs with an average length 570.7 bp and N50 size of 806 bp were designated as the channel catfish testis transcriptome in the following steps of analysis (Table 2).

**Table 2:** Assembly statistics of the testis transcriptome using Trans-ABySS.

Assembly Metrics	Number
No. of assembled contigs from ABySS & TransABySS	5,977,085
No. of contigs after removing redundancy	193,462
Average length of final testis transcriptome (bp)	570.7
Final N50 (bp)	806
Total number of known unigenes	25,307
Total number of newly identified catfish unigenes	167

### Gene Identification and Annotation of Channel Catfish Testis Transcriptome

Gene identification of the assembled contigs was performed using BLASTX searches against the reference proteins available at NCBI zebrafish RefSeq and Uniprot protein databases to annotate the channel catfish testis transcriptome. After annotation, approximately 68,000 assembled contigs had hits in non-redundant database. A total of 55,521 assembled contigs had significant (E-value ≤1e-10) hits to the zebrafish RefSeq database, corresponding to 17,180 unique proteins. In searches against the Uniprot database, 47,383 catfish contigs had significant hits, accounting for a total of 21,877 unique proteins. Cumulatively, a total of 67,923 catfish contigs had at least one significant hit against the two databases, allowing 25,307 unigenes to be identified in channel catfish testis transcriptome (Table S1).

**Figure 1:**
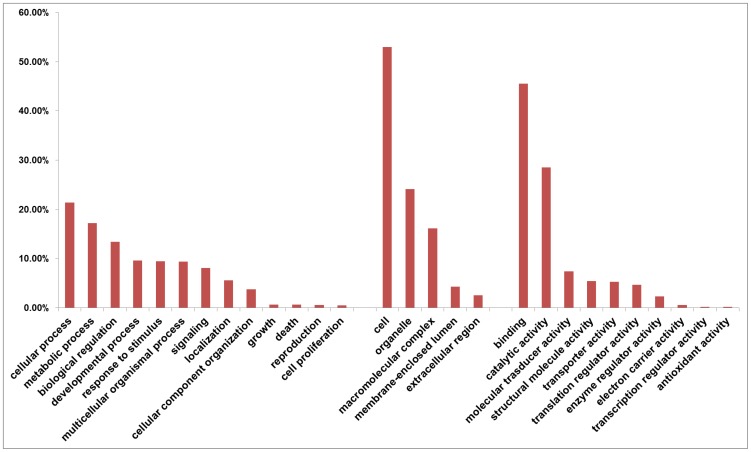
GO term classifications of channel catfish testis transcriptome. GO annotations were based on zebrafish RefSeq and GO-terms were processed by Blast2Go and categorized at level 2 into three major functional categories (biological process, cellular component, and molecular function).

Unique gene-coding contigs from the channel catfish testis reference assembly were then used as inputs to perform gene ontology (GO) annotation by Blast2GO [37]. A total of 28 GO terms including 13 (46.4%) cellular component terms, 5 (17.9%) molecular functions terms and 10 (35.7%) biological process terms were assigned to 25,307 unique gene matches. The percentages of annotated catfish sequences assigned to GO terms are shown in Figure 1. Analysis of level 2 GO term distribution showed that cell (GO:0005623), binding (GO:0005488), and cellular process (GO:0009987) were the most common annotation terms in the three GO categories (Figure 1).

### Newly Identified Genes in Catfish

To identify transcripts that were newly identified from the testis, all assembled contigs from testis transcriptome were used as queries to perform BLASTN searches against the existing catfish transcriptome. A total of 335 unique novel contigs from testis transcriptome had no blast hits against the existing catfish transcriptome assemblies. As more than one contigs had hits to a single unique gene, these 335 contigs corresponded to 167 putative unique novel genes when searched against the NR database using BLASTX (Table S2), suggesting that 167 genes were newly identified from the catfish testis.

### Identification and Distribution of the Putative Male-biased Genes

In order to identify male-biased genes, i.e., genes that are preferentially expressed in the male, a meta-analysis of RNA-Seq data was conducted to compare expression ratios in the testis (male) and in the gynogen (female). The gynogen was a doubled haploid fish that are homozygous without the Y-chromosome [38]. *In silico* mapping of RNA-Seq reads allowed 81% high quality sequencing reads of the testis RNA-Seq to be mapped onto the testis transcriptome, while 71% high quality sequencing reads from the gynogen catfish RNA-Seq [33] were mapped onto the testis transcriptome. Using a 5-fold ratio cut-off (expressed five times more in male than in the female), a total of 5,450 genes were found to exhibit preferential expression in the testis. Of these, 637 genes were expressed 30-fold or more in testis than in females, 1,897 genes were expressed 10–30 fold more in testis than in the gynogen, and 2,916 genes were expressed 5–10 fold more in the testis than in the gynogen (Table S3).

**Figure 2:**
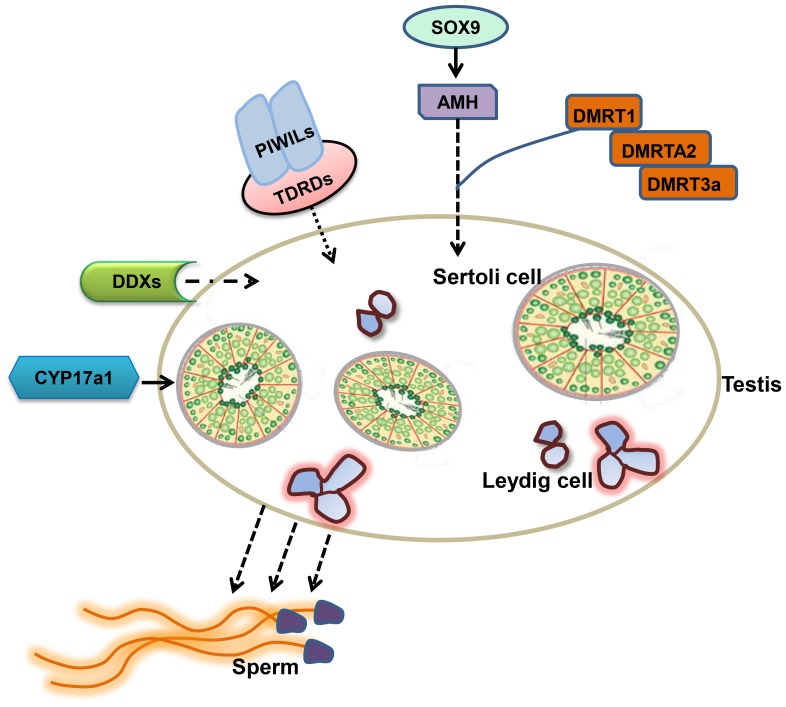
Putative catfish spermatogenic pathway based on RNA-Seq expression signatures in channel catfish testis.

BLASTX analysis indicated that many male-biased genes were involved in gonadogenesis, spermatogenesis, testicular determination, gametogenesis, gonad differentiation and sex determination (Table 3). Some examples of these genes include doublesex- and mab-3-related transcription factor 1 isoform 1 (Dmrt1, the sex-determining gene in medaka), Dmrta2, Dmrt3a, Amh (Amhr2, probably responsible for sex determination in fugu; amhy, a strong candidate sex-determining gene in Patagonian pejerrey), Ddx4, Ddx11, and transcription factor Sox9. Gene pathway analysis indicated that many of these genes are involved in the regulation of spermatogenesis (Figure 2).

**Table 3:** Representative channel catfish male-biased genes involved in spermatogenesis, gonad sex determination, and testicular determination.

Feature ID	Gene name in zebrafish database	Abbreviations	Fold change	Linkage group
Contig29101	Piwi-like protein 1	PIWIL1	33.9	2
Contig22821	Transcription factor SOX-9	SOX9	7.2	3
Contig20336	IQ motif containing H	IQCH	48.8	4
Contig1684	Kinesin family member 23	KIF23	12.5	4
Contig24841	DEAD/H (Asp-Glu-Ala-Asp/His) box helicase 11	DDX11	12.2	4
Contig21605	HYDIN, axonemal central pair apparatus protein	HYDIN	31.1	4
K70∶510778	Transforming growth factor beta regulator 1	TBRG1	9.3	4
Contig24257	Aquaporin 11	AQP11	7.3	4
Contig21219	Nucleoporin 93	NUP93	5.5	4
Contig17653	Cytochrome P450, family 17, subfamily A, polypeptide 1	CYP17A1	24.0	6
Contig25669	Doublesex- and mab-3-related transcription factor A2	DMRTA2	504.2	8
Contig5339	Polyadenylate-binding protein 1-like	PABPC1	614.2	11
Contig23715	Deleted in azoospermia-like	DAZL	702.4	12
Contig11277	Spermatogenesis-associated protein 22-like	SPATA22	369.3	14
Contig27135	Spermatogenesis-associated protein 17	SPATA17	50.8	16
Contig17343	Ring finger protein 17	RNF17	355.9	17
Contig22169	Anti-Mullerian hormone	AMH	11	20
Contig19377	Glutamine-dependent NAD(+) synthetase	NADSYN1	10.0	20
Contig30394	Tektin-1	TEKT1	94.4	22
Contig19309	Piwi-like protein 2	PIWIL2	362	25
Contig30210	Probable ATP-dependent RNA helicase DDX4	DDX4	146.3	25
Contig17400	Testis-specific gene 10 protein	TSGA10	265.6	26
Contig18399	Doublesex- and mab-3-related transcription factor 1 isoform 1	DMRT1	1009.5	N/A
Contig11347	Doublesex and mab-3 related transcription factor 3a	DMRT3a	50.3	N/A
Contig26052	Serine/threonine-protein kinase Nek1	NEK1	8.0	N/A
Contig9056	Protein arginine N-methyltransferase 5	PRMT5	5.6	N/A
Contig22278	RuvB-like 2	RUVBL2	8.9	N/A
Contig17332	Sperm-associated antigen 17	SPAG17	74.4	N/A
Contig6953	Sperm-associated antigen 8-like	SPAG8	42.4	N/A
Contig20873	Spermatogenesis-associated protein 4	SPATA4	37.6	N/A
Contig21931	Transforming acidic coiled-coil-containing protein 3	TACC3	5.1	N/A
K94∶490875	Tudor domain containing 1	TDRD1	894.3	N/A
Contig23678	Tudor domain containing 7 isoform 1	TDRD7	404.6	N/A
Contig23675	Testis-expressed sequence 9 protein	TEX9	36.4	N/A
Contig25594	Testis-expressed sequence 14 protein	TEX14	325.8	N/A

**Figure 3:**
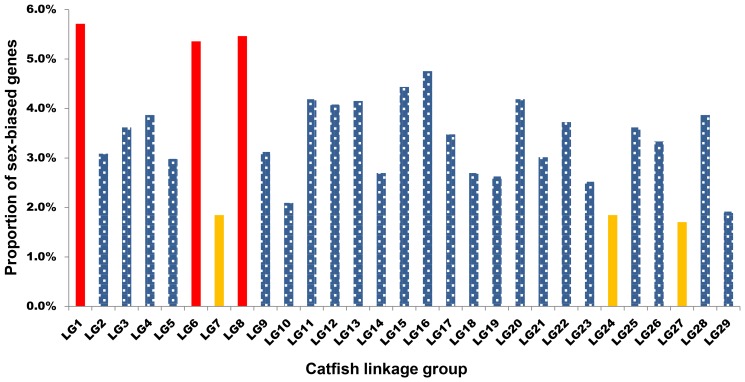
Linkage group distribution of the male-biased genes. Note that the proportion of male-biased genes per linkage group is similar except for chromosomes 1, 6, 8, 7, 24 and 27 (shown in red for linkage groups with high numbers of male-biased genes and in yellow for linkage groups with low numbers of male-biased genes).

Genomic locations of the male-biased genes were analyzed by sequence homology searches for their associated genomic draft sequence contigs. If the linkage group information is known for their associated genomic contigs, then by inference, their genomic locations are known. Analysis of genomic locations of the male-biased genes indicated that they are distributed all over the genome among all 29 linkage groups (Figure 3). Of the 5,450 male-biased genes, 2,818 genes could be assigned to linkage groups, whereas the remaining 2,632 genes could not be assigned to linkage groups. As shown in Figure 3, the distribution of the male-biased genes is on all linkage groups. However, when the genes mostly related to spermatogenesis, gonad differentiation, and sex determination were analyzed, quite a few genes were located in linkage groups 4 (Table 3), which included the sex determination locus in catfish [39]. Blast2GO program revealed a total of 31 GO terms including 7 cellular component terms, 11 molecular functions terms and 13 biological process terms. An overview of the number and the proportion of the annotated genes assigned to GO terms are shown in Figure S1.

## Discussion

In this study, we conducted RNA-Seq analysis of the catfish testis that allowed expression profiling of genes expressed in the male-specific organ. In this process, over 25,307 unigenes were identified from the catfish testis, of which 167 unigenes were identified for the first time in catfish. This RNA-Seq analysis enhanced the transcriptome assembly in catfish, and will support the whole genome annotation of catfish.

Our long term goal is to determine the sex determination mechanisms in catfish. In addition to transcriptome profiling, we were interested in exploring the possibility of identifying male-biased genes. We therefore compared the transcriptome expression profiles in the testis to those of the gynogen female catfish. The gynogen female, a doubled haploid female, has two identical copies of each chromosome but without the Y-chromosome [38]. For the construction of the gynogen female transcriptome [29], 19 tissues were collected from a single doubled haploid female channel catfish adult, including head kidney, fin, pancreas, spleen, gill, brain, trunk kidney, adipose, liver, stomach, gall bladder, ovary, intestine, thymus, skin, eye, swim bladder, muscle, and heart. However, no testis tissue was included because the female fish does not harbor the male-specific organ of testis. Therefore, this RNA-Seq conducted with testis tissue should enhance the transcriptome assemblies by adding on testis-specific or preferentially expressed genes.

A large number of genes were identified to express significantly higher in the male testis than in the gynogen fish. Overall, more than 5,000 had an expression ratio of greater than 5 fold in the testis than in the gynogen, however, only 637 genes were expressed much higher (>30 times) in the testis than in the gynogen fish. These putative male-biased genes can be interesting because they could be candidates contributing to gonadogenesis or testicular development and differentiation, or even sex determination. However, we realized the limitation of meta-analysis for the comparison of expression in different sexes. On the one hand, the genes important for sex differentiation are likely to be expressed in sex-specific organs such as the testis under this study. We understand that there is no direct control for such experiments because only male fish have testis. Therefore, we accepted the compromise in this initial study in order to more broadly capture the male-biased genes. In a way, the putative male-biased genes identified here can only serve as proxies of sex-biased genes. Nonetheless, a large number of genes with identities to known genes involved in gonadogenesis, spermatogenesis, testicular development and differentiation and sex determination were identified among the male-biased genes (Table 3). Interestingly, many spermatogenesis genes were believed to accumulate on the Y chromosome, suggesting their potential location in the sex-determining region [40]. Among many of the male-biased genes, a number of them are highly relevant to sex determination and differentiation, and here we discuss a few:

### Dmrt Genes

Of great interest in this study was the detection of the extremely high fold ratio in testis for three genes Dmrt1, Dmrta2 and Dmrt3a belonging to the Dmrt gene family. Characterized by a conserved DNA-binding motif known as the Doublesex- and Mab-3-related (DM) domain, Dmrt genes have been reported to be actively involved in sex determination and/or differentiation. These genes stimulate male-specific differentiation, but repress female-specific differentiation [41]–[43]. In medaka, Dmrt1 on the Y-chromosome is the master sex determination gene. The expression pattern of Dmrt genes has been studied and is clearly consistent with such related functions in southern catfish *Silurus meridionals*, North African catfish *Clarias gariepinus*, and *Danio rerio*, suggesting the importance of this gene family during gonad development, testis sex determination, and testis differentiation [44]–[48]. In the report of Siberian sturgeon, Dmrt1 showed a significantly higher expression in testis. Similar trend of dmrt1 expression during early and advanced stages of gonad development was observed in sturgeons [49], as well as in rainbow trout [50]. Dmrta2, regarded as one of the candidate genes related to sex determination and gonad differentiation, was mapped onto the linkage group containing the major sex determination factor in turbot [41]. Even though Dmrta2 was not the primary sex determination gene, it was thought to be involved in the sexual development of turbot [51]. The higher level of expression of Dmrt3a, which expressed in the same temporal and spatial pattern as Dmrt1, suggests its putative role in the developing gonads and sex determination [52], [53]. In this study, we identified Dmrt1, Dmrta2, and Dmrt3a among the male-biased genes. Although their function in channel catfish is unknown at present, their involvement in sex determination and differentiation needs to be further studied.

### TDRDs and PIWIs

TDRD1 and TDRD7, both essential proteins for spermatogenesis, were found to be male-biased genes in this study. They were previously shown to be preferentially expressed in the murine testis [54], and function during spermatogenesis [55]. Each member in the TDRD gene family performs a distinct function at different differentiation stages of spermatogenesis and TDRD7 was demonstrated to play a crucial role during early spermatid differentiation [56].

In this study, we also identified PIWIL1 and PIWIL2 as male-biased genes. PIWIL1 and PIWIL2 are members of the mouse Piwi family proteins (MIWI, MILI, and MIWI2) that play important roles in spermatogenesis through transcriptional and post-transcriptional regulation. Acting as a functional partner of Piwi family proteins, TDRD group of Tudor proteins were reported to be physiological binding partners of Piwi family proteins and coordinately work together in the regulation of spermiogenesis [54], [57]. Here in our findings, the fact that both PIWIs and TDRDs were identified among the male-biased genes is intriguing, suggesting that they may similarly work side by side in coordination of spermatogenesis. Further investigations are needed to gain understanding of the functions of these genes.

### DDXs

DDX4 and DDX11 exhibited high level of expression in the catfish testis, and were both identified as male-biased genes. DDX4 and DDX11, both members of the DEAD/DEAH box family of helicases, were believed to be involved in embryogenesis, spermatogenesis, and cellular growth and division [58]. DDX4, as one of the 14-3-3 interactors predominantly expressed in testis, was reported to be essential for spermatogenesis due to its importance in germ cell division and maturation [59]. In another study, DDX4 (the gene encoding Drosophila VASA homolog) was shown to play an essential role in regulating germ cell differentiation in both vertebrates and invertebrates [60]bib20. In humans, DDX4 mRNA and protein were abundantly and specifically expressed in germ cells in both sexes throughout development [61]bib33, suggesting a role in germ cell development. Rolland et al. performed *in situ* hybridization and detected very strong signal for DDX4 in rainbow trout testis, confirming its function in spermatogenesis regulation [62]. DDX11 was shown to be expressed ubiquitously during early embryogenesis and act as one of the necessary proteins for the later stages of spermatogenesis in the mouse testis [63], [64]. Once again, the exact function of DDX4 and DDX11 in catfish is unknown at present, but certainly worthwhile of additional study.

### Sox9

Interestingly, Sox9, one of the most important genes expressed during testis determination in mammals [65], was found to be highly expressed in the catfish testis, and as a male-biased gene. Previous studies about Sox9 in mice [66]–[69] and frogs [70], [71] indicated its critical role in testis differentiation and development. Sox9, together with Dmrt1, is one of the key SRY targets in mammalian testis development. In mice, Sox9 was also considered to be required and sufficient for testis formation [72]. Sox9 was also reported to be involved in vertebrate sex determination [73]. In the male sex-determination pathway, Sox9 is a very early-acting gene. For instance, mutations of Sox9 have been shown to interfere with male sex determination, suggesting its role in testis determination [74]. Much fewer studies were conducted with Sox9 in fish. It was conserved and expressed during testicular development stage in fish, and was believed to be a candidate gene involved in testis differentiation, but not in sex determination [49], [75]. Apparently, as the male-biased gene in catfish, the functions of Sox9 in catfish testis development need to be studied.

## Materials and Methods

### Ethics Statement, Experimental Fish and Sample Collection

All procedures involving the handling and treatment of fish used during this study were approved by the Auburn University Institutional Animal Care and Use Committee (AU-IACUC) prior to initiation of experiments. The fish used for this project were 25 male channel catfish including 13 one-year-old juveniles, 8 two-year-old adults and 4 four-year-old sexual maturation adults. All fish were sexed based on external genitalia, followed by anatomical confirmation. The fish were euthanized with tricaine methanesulfonate (MS 222) at 300 mg/L (buffered with sodium bicarbonate) before sample collection. Testis tissues from the 3 different ages were placed into 5 mL RNA later™ (Ambion, Austin, TX, USA) respectively. After one day of temporary storage at 4°C, samples immersed in the RNA later™ were transferred to a −80°C ultra-low freezer until preparation of RNA.

### RNA Isolation, Library Construction and Illumina Sequencing

Prior to RNA extraction, samples were removed from the −80°C freezer and ground with sterilized mortar and pestle in the presence of liquid nitrogen to a fine powder. Total RNA was extracted from testis powder using the RNeasy Plus Kit (Qiagen) treated with RNase free DNase I (Qiagen) to remove genomic DNA. RNA concentration and integrity was measured on an Agilent 2100 Bioanalyzer using a RNA Nano Bioanalysis chip. Equal amount of total RNA from catfish testis of each age was pooled together into only one sample for use in RNA-Seq.

RNA-Seq library preparation and sequencing was carried out by HudsonAlpha Genomic Services Lab (Huntsville, AL, USA) as previously described by Li et al. and Sun et al. [36], [76]. cDNA library was prepared with ∼4 µg of starting total RNA following the protocols of the Illumina TruSeq RNA Sample Preparation Kit (Illumina). The library was amplified with 15 cycles of PCR. The final library had an average fragment size of ∼270 bp and final yields of ∼400 ng. After KAPA quantitation and dilution, the library was sequenced with one lane on an Illumina HiSeq 2000 instrument with 100 bp paired end (PE) reads. Raw read data of channel catfish testis RNA-Seq are archived at the NCBI Sequence Read Archive (SRA) under Accession SRP018265 and now released.

### *De novo* Assembly of Sequencing Reads

Before *de novo* assembly, raw sequencing reads were trimmed by removing adaptor sequences, ambiguous nucleotides (‘N’ at the end of reads), low quality sequences (quality score less than 20), and short read length sequences (length below 30 bp) with CLC Genomics Workbench (version 4.8; CLC bio, Aarhus, Denmark) as previously described [36], [76]. The assembly was performed using the *de Brujin* graph approach with ABySS (version 1.3.4) [77] and Trans-ABySS version 1.3.2 to obtain accurate and reliable consensus contigs as a reference assembly [78]. Briefly, continuous multiple k-mers ranging from 50 to 96 were used in ABySS, and then all 47 assemblies from ABySS were merged into one assembly to generate the transcriptome assembly using Trans-ABySS. Afterwards, CAP3 [79] was utilized in order to minimize redundancy and the resulting contigs that were >200 bp were regarded as final non-redundant transcripts. The threshold was set as 100 bp for the minimal overlap length and 99% for the identity in CAP3.

### Transcriptome Annotation and Ontology

The assembled contigs including both unique transcripts and singletons were used as queries for BLAST searches against the zebrafish RefSeq protein database, UniProtKB/SwissProt database and non-redundant database, respectively. Searches were conducted using the BLASTX program, with an E-value cut-off of 1e-10 and matching to the top hits. Gene ontology (GO) annotation analysis was performed using the zebrafish BLAST results in Blast2GO version 2.5.1, which is an automated tool for the assignment of gene ontology terms. The zebrafish BLAST result was imported to Blast2GO. The final annotation file was produced after GO-mapping, GO term assignment, annotation augmentation and generic GO-Slim process. The annotation result was categorized with respect to Biological Process, Molecular Function, and Cellular Component at level 2.

### Identification of Putative Novel Transcripts

In order to identify putatively novel transcripts from the currently assembled testis transcriptome, the assembled contigs were used as queries to search against the existing catfish transcriptome databases [33], [36], [76], using blast with E-value cutoff of 1e-5. Those transcripts without blast hits after *in silico* substraction were identified as putative newly identified transcripts.

### Identification of the Male-biased Genes

To identify the sex-biased transcripts, high quality sequence reads from the male catfish testis RNA-Seq reads (current work) and the female doubled haploid catfish RNA-Seq reads [33] were mapped onto the testis transcriptome using CLC Genomics Workbench respectively. At least 95% of the read length was required to align to the reference and a maximum of two mismatches were allowed during mapping. The unique gene reads number for each transcript was determined, and then normalized to RPKM (Reads Per Kilobase per Million). The proportions-based Kal’s test for differences was used to identify the differentially expressed genes between testis and the gynogen female with p-value <0.05. The fold changes were calculated after quantile normalization of the RPKM values. Transcripts with absolute fold change values of larger than 5 and total read number larger than 10 were included in analysis as the sex-biased genes.

Gene ontology (GO) annotation analysis was performed on the preferentially expressed genes in testis using Blast2GO version 2.5.1. The final annotation file was produced after GO-mapping, GO term assignment, annotation augmentation and generic GO-Slim process. The annotation result was categorized with respect to Biological Process, Molecular Function, and Cellular Component at Level 2.

In order to determine the genome distribution, the male-biased genes were mapped to the catfish linkage groups by taking advantage of the existing genomic resources, especially the integrated genetic linkage and physical map [80].

## Supporting Information

Figure S1 GO annotations of preferentially expressed genes in the testis
(TIF)

Table S1 Catfish testis transcriptome contigs and gene annotation.
(XLSX)

Table S2 Putative novel identified transcripts identified by channel catfish testis RNA-Seq.
(XLSX)

Table S3 Sex-biased genes detected by RNA-Seq analysis of channel catfish testis transcriptome
(XLSX)
