# Supplementary figures and images for: Male-Biased Genes in Catfish as Revealed by RNA-Seq Analysis of the Testis Transcriptome
Source: PLoS One. 2013 Jul 12;8(7):e68452. doi: 10.1371/journal.pone.0068452 (PMC3709890; doi:10.1371/journal.pone.0068452)

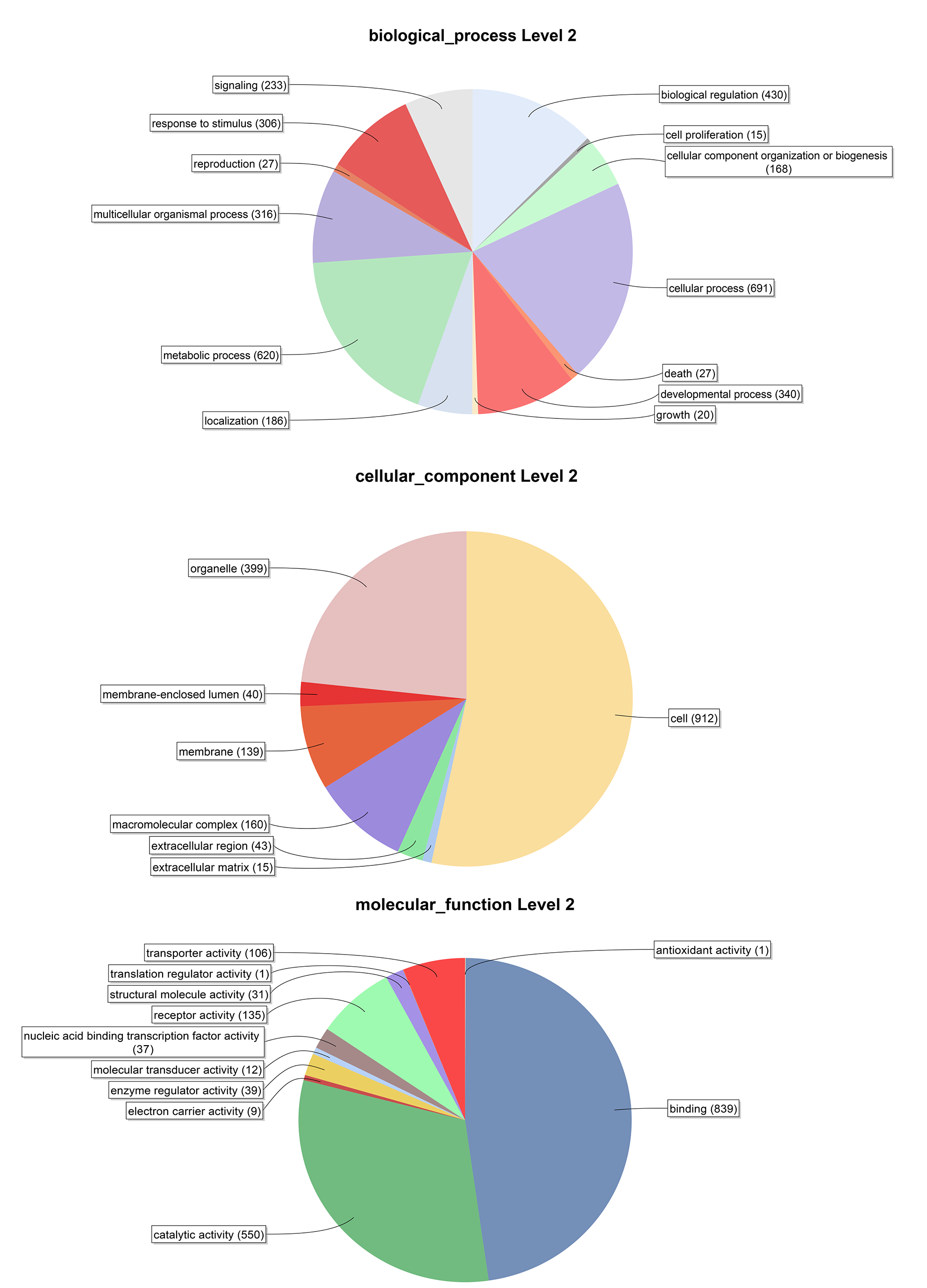

Supplement: Figure S1 — GO annotations of preferentially expressed genes in the testis (TIF) [file pone.0068452.s001.tif]
